# Dataset on UAV RGB videos acquired over a vineyard including bunch labels for object detection and tracking

**DOI:** 10.1016/j.dib.2022.108848

**Published:** 2022-12-23

**Authors:** Mar Ariza-Sentís, Sergio Vélez, João Valente

**Affiliations:** Information Technology Group, Wageningen University & Research, 6708 PB Wageningen, the Netherlands

**Keywords:** Viticulture, Precision agriculture, Object detection, Object tracking, Remote sensing, UAV

## Abstract

Counting the number of grape bunches at an early stage of development offers relevant information to the winegrower about the potential yield to be harvested. However, manual counting on the fields is laborious and time-consuming. Remote sensing, and more precisely unmanned aerial vehicles mounted with RGB or multispectral cameras, facilitate this task rapidly and accurately. This dataset contains 40 RGB videos from a 1.06-ha vineyard located in northern Spain. Moreover, the dataset includes mask labels of visible grape bunches. The videos were acquired throughout four UAV flights with an RGB camera tilted at 60 degrees. Each flight recorded one side of a row of the vineyard. The grape berries were between pea-size (BBCH75) and bunch closure (BBCH79) stage, which is two months before harvesting. No operations other than those usual in a commercial vineyard, such as pruning, cane tying, fertilization, and pest treatment, have been carried out, hence, the dataset presents leaf occlusion. The dataset was gathered and labelled to train object detection and tracking algorithms for grape bunch counting. Furthermore, it eases the work of winegrowers to check the sanitary status of the vineyard.

**Specifications Table**

**Table:** 

Subject	Agricultural Sciences, Agronomy and Crop Science
Specific subject area	Object detection in agriculture using UAVs
Type of data	Video and annotations in PNG format
How the data were acquired	Unmanned aerial vehicle: DJI Matrice 210 RTK (DJI Sciences and Technologies Ltd., Shenzhen, Guangdong, China). Flight speed: 0.7 m/s Flight altitude: 3 m AGL Sensor: DJI Zenmuse X5S Sensor characteristics: focal aperture range: f1.7 - f.16, shutter speed: 1/8000. Video characteristics: frame width: 4096, frame height: 2160, frame rate: 59.94 frames/second. A total of four flights were executed. Each flight recorded one row of the vineyard, whose length is around 110 m.
Data format	Raw
Description of data collection	The four flights were executed on June 28^th,^ 2021 over four rows of the vineyards. The flights were carried out on a sunny day with wind velocity lower than 0.5 m/s. The four rows were selected according to the ripening stage of the grape clusters, to have a representative sample of the development status over the four rows. The grape bunch annotations were labelled using the CVAT software and are available in the MOTS style [4].
Data source location	Institution: Wageningen University & Research City/Town/Region: Tomiño, Pontevedra, Galicia Country: Spain Latitude and longitude (and GPS coordinates) for collected samples/data: 41°57′18.3″N 8°47′41.9″W
Data accessibility	Repository name: Zenodo Data identification number: 10.5281/zenodo.7330951 Direct URL to data: https://zenodo.org/record/7330951#.Y3tU3nbMKUk
Related research article	Ariza-Sentís, M., Vélez, S., Baja, H., & Valente, J. (2022). *IPPS 2022 Conference Book*. 231.

## Value of the Data

• Dataset is useful for researchers interested in instance segmentation, as it allows the detection and tracking of the clusters [2].
• Dataset can be employed to count the number of visible grape clusters per row, which can provide insights into the potential yield to be harvested.
• Dataset eases the work for winegrowers to visually check the sanitary status of the vineyard.
• Data is of importance for researchers with an interest in phenotyping since phenotyping traits such as the bunch length and width can be extracted.

## 1 Objective

The main purpose of this dataset is to acquire RGB videos of the side of vineyard rows and the labels of the bunches to provide enough information to analyze 1) the application of object detection and tracking algorithms in vineyards for grape cluster detection and tracking, and 2) the counting of the number of visible grape clusters per row for early yield assessment.

## 2 Data Description

**Fig. 1:**
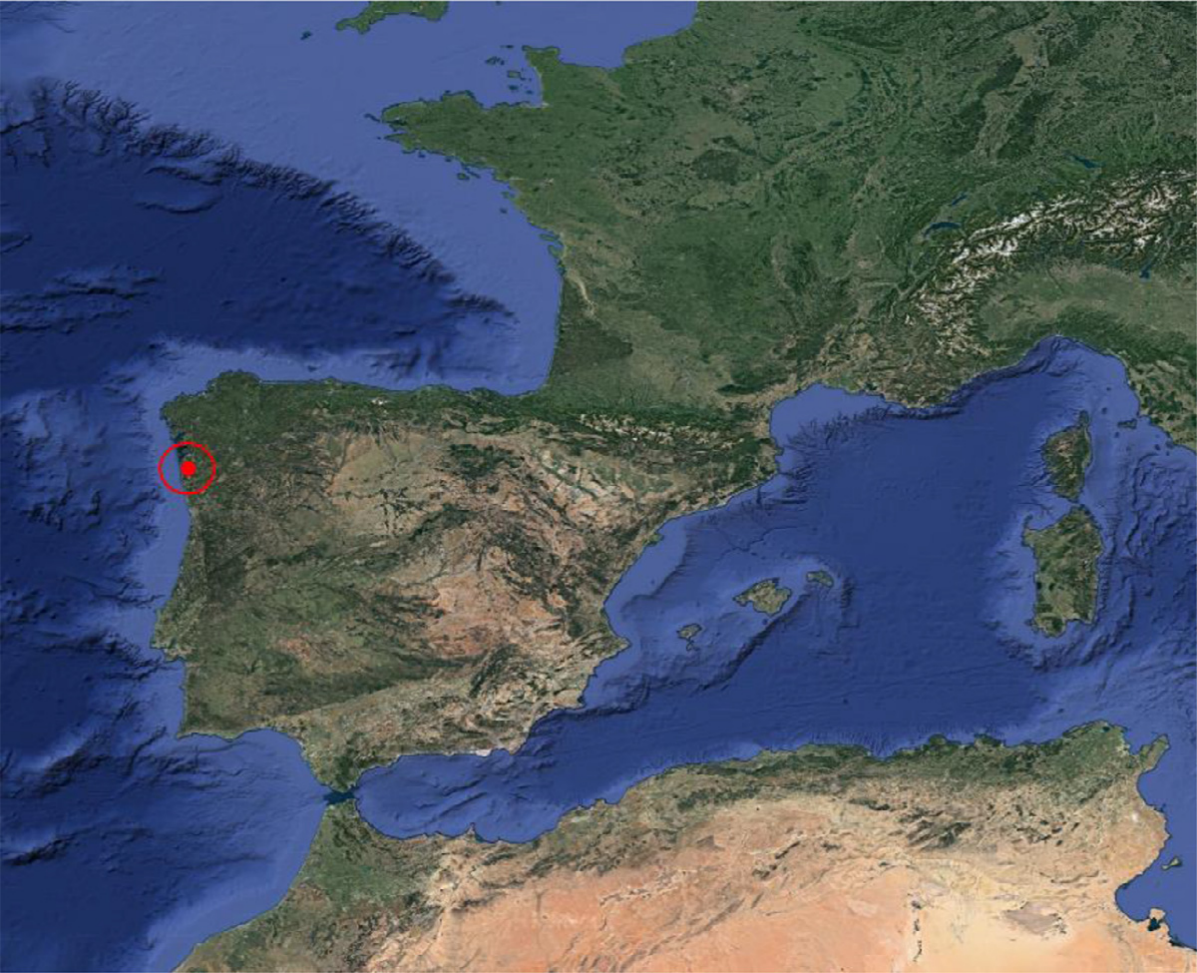
Location of the vineyard over the Iberian peninsula. Coordinates: 41°57′18.3″N 8°47′41.9″W (WGS84).

**Fig. 2:**
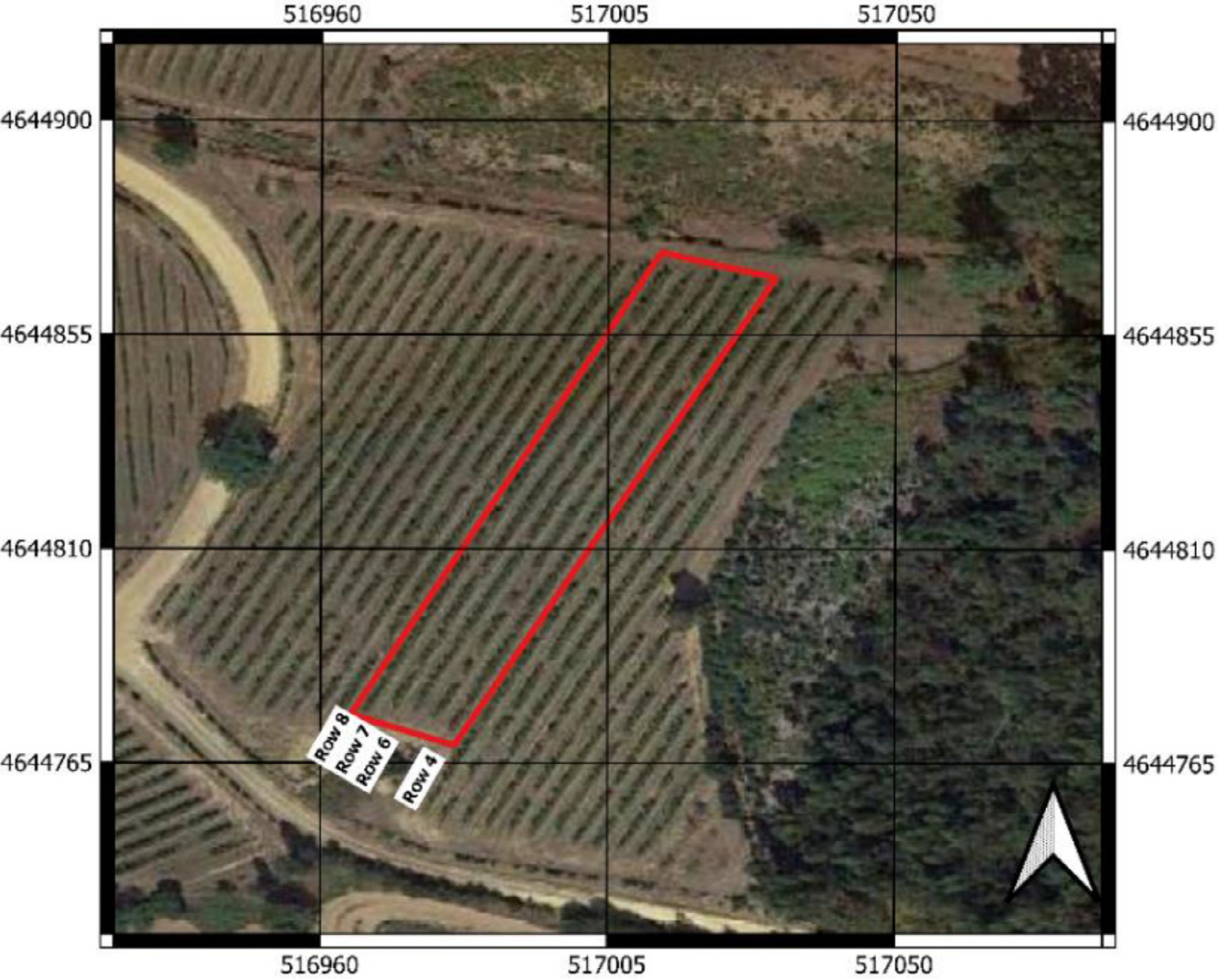
Location of rows 4, 6, 7, and 8 in the vineyard. Coordinates in ETRS89 / UTM zone 29 N.

The flights were executed during the 2021 campaign, on June 28th, over four rows of a 1.06-ha commercial vineyard *Vitis vinifera* cv. Loureiro. The vineyard is located in Tomiño, Spain, and belongs to 'Bodegas Terras Gauda, S.A.' (X: 516989.02, Y: 4644806.53; ETRS89 / UTM zone 29 N) (Figs. 1 and 2). The plants were planted in 1990 with a NE-SW orientation and trained in vertical shoot positioning and managed in vertical trellis system. The vines are managed using a spur-pruning system with 3 to 6 positions per plant. The distance between plants and rows is 2.5  × 3 m, respectively. Spontaneous vegetation species grow between rows. A 196.17C rootstock was chosen to graft the plants since that rootstock is resistant to active limestone and is suitable for soils with excessive humidity, as is common in this region. The management operations carried out during the year are the common ones in commercial vineyards, which are pruning, cane tying, fertilization, and pest treatment. No leaf removal was carried out, hence, the dataset presents leaf occlusion.

### 2.1 RGB videos

The dataset [1] includes a total of 40 RGB videos and grape cluster annotations collected during four flights. Table 1 presents the number of videos acquired per flight. The names of the videos are structured as “Row*rowNumber*.*videoclipNumber*_*partNumber*”. For instance, Row4.1_1 refers to the first video clip recorded for row 4, part 1. Therefore, all videos that have the same row number and video clip number are contiguous, starting from part 1 until the last part number. As can be observed in Table 1, there is no row 5, which was not recorded since there were many plants affected by esca disease and thus not many grape clusters were present. Since no operations other than those usual in a commercial vineyard have been carried out, and no leaf removal is performed, the dataset presents leaf occlusion, which can be of interest to researchers to study the detection of half-hidden clusters.

**Table 1:** Number of videos recorded per flight and row. The total size of the videos recorded per row (in GB) sums up to 7.5 GB of data.

Flight number	Vineyard row	Number of videos	Total size of the videos (GB)
1	4	14	2.01
2	6	8	0.99
3	7	14	3.47
4	8	4	1.02

### 2.2 Annotation procedure

A total of 29 videos of the vineyard were annotated for grape cluster detection and tracking using the CVAT software, an annotation tool that was developed by Intel. The videos used the MOTS style [4]. The MOTS annotations are temporally consistent, meaning that each grape cluster instance is consistent throughout the video sequence. The grape clusters were annotated with a per-pixel accuracy, making sure that only the grapes were annotated, without the peduncle of the cluster. A grape cluster was annotated if it was visible on the camera, even when it was under a shade.

**Fig. 3:**
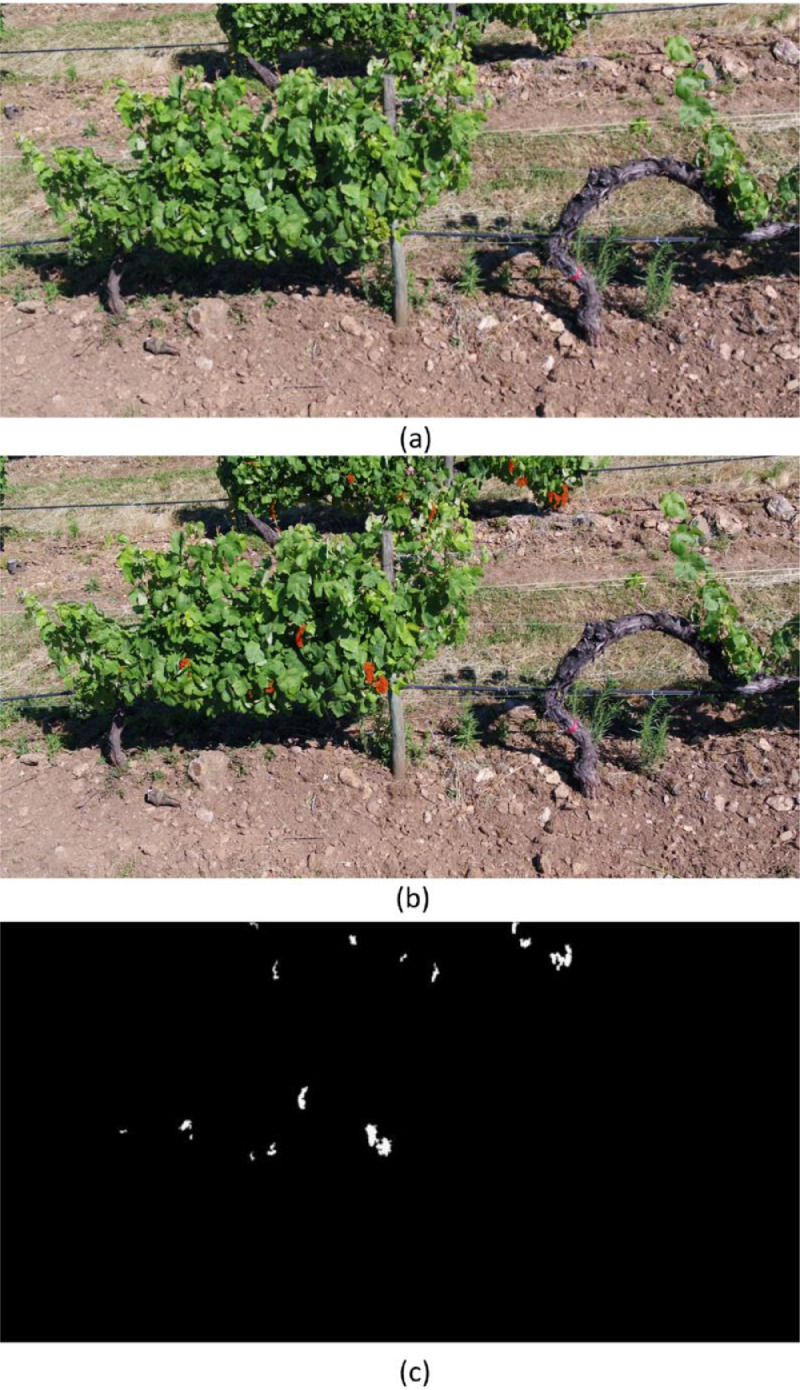
Snapshot of Row 6.1_3. (a) Input frame, as recorded with the UAV. (b) Instance overlay. Each red mask is a grape cluster annotation as labelled by the user. (c) Annotation output, in white, on a black background. The total number of visible grape clusters in the snapshot is 16.

The annotations are available in PNG format. The number of frames per video ranges from 15 to 28, with a total number of 681 labelled frames. Fig. 3 shows an example of how the grape clusters present in Row 6.1_3 were annotated. To provide an example of how object detection can be performed using this dataset, all the grape bunches observed (not hidden because of leaf occlusion) have been annotated with red masks. This dataset provides cluster masks of 29 videos to train object detection and/or tracking algorithms, for instance, Mask R-CNN [3] or PointTrack [5].

## 3 Experimental Design, Materials and Methods

The flights were carried out on June 28th 2021 over a 1.06-ha vineyard, *Vitis vinifera* cv. Loureiro, using a multi-rotor platform (DJI Matrice 210 RTK). The flight conditions were a clear sky and wind speed lower than 0.5 m/s. The videos were recorded at 3 m above ground level, just above the rows of the vineyard, to get an accurate and close overview of the plants and the grape clusters.

The camera (DJI Zenmuse X5S) has a 20.8-megapixel sensor that captures images at a high resolution of 3.4 μm pixel size. The frame width and height of the recorded videos are 4096 × 2160, respectively.

In the matter of not reducing the research possibilities and further analysis of the dataset, no processing of the acquired videos is done and hence, the videos are as taken by the RGB sensor.

## Ethics Statements

The authors state that the present work meets the ethical requirements for publication in Data in Brief. The work does not involve studies with animals and humans.

## CRediT authorship contribution statement

**Mar Ariza-Sentís:** Investigation, Methodology, Data curation, Visualization, Writing – original draft. **Sergio Vélez:** Investigation, Methodology, Data curation, Writing – review & editing. **João Valente:** Conceptualization, Supervision, Writing – review & editing.

## Declaration of Competing Interest

The authors declare that they have no known competing financial interests or personal relationships that could have appeared to influence the work reported in this paper.

## Data Availability

Early Botrytis in Terras Gauda 2021 (Original data) (Zenodo)
